# Can Systems Biology Advance Clinical Precision Oncology?

**DOI:** 10.3390/cancers13246312

**Published:** 2021-12-16

**Authors:** Andrea Rocca, Boris N. Kholodenko

**Affiliations:** 1 Hygiene and Public Health, Local Health Unit of Romagna, 47121 Forlì, Italy; 2 Systems Biology Ireland, School of Medicine, University College Dublin, Belfield, D04 V1W8 Dublin, Ireland; 3 Conway Institute of Biomolecular and Biomedical Research, University College Dublin, Belfield, D04 V1W8 Dublin, Ireland; 4 Department of Pharmacology, Yale University School of Medicine, New Haven, CT 06520, USA

**Keywords:** cancer systems biology, statistical methods, network analysis, mathematical models, signaling networks, drug resistance, patient-specific network modeling, precision oncology

## Abstract

**Simple Summary:**

Omics technologies and targeted drugs are revolutionizing the clinical oncology landscape, portending the promise of precision oncology. However, our abilities to define the best treatment for the individual tumor, based on its molecular characterization, are still limited. Systems biology, by studying the collective behavior of the different types of molecules involved in a biological process, allows us to reconstruct the complex behavior of biological systems and to compute the system’s response to perturbations, such as targeted therapies. This helps to dissect drug resistance phenomena, as well as to establish the best drug combinations for a specific tumor. Patient-specific biomarkers can be built from dynamical models of signaling networks and can have a greater prognostic value than conventional biomarkers. In this paper, we review current systems biology methods to highlight how they can contribute to advance clinical and translational research in precision oncology.

**Abstract:**

Precision oncology is perceived as a way forward to treat individual cancer patients. However, knowing particular cancer mutations is not enough for optimal therapeutic treatment, because cancer genotype-phenotype relationships are nonlinear and dynamic. Systems biology studies the biological processes at the systems’ level, using an array of techniques, ranging from statistical methods to network reconstruction and analysis, to mathematical modeling. Its goal is to reconstruct the complex and often counterintuitive dynamic behavior of biological systems and quantitatively predict their responses to environmental perturbations. In this paper, we review the impact of systems biology on precision oncology. We show examples of how the analysis of signal transduction networks allows to dissect resistance to targeted therapies and inform the choice of combinations of targeted drugs based on tumor molecular alterations. Patient-specific biomarkers based on dynamical models of signaling networks can have a greater prognostic value than conventional biomarkers. These examples support systems biology models as valuable tools to advance clinical and translational oncological research.

## 1. Introduction

Research in clinical oncology has always been confronted with the limited efficacy of single drugs and the need to identify the best treatment for a specific patient. Yet, the cornerstone of clinical cancer research, phase 3 randomized controlled trials (RCTs), aims to define the average treatment effect in a population of patients [1]. Not surprisingly, criticisms have emerged, questioning whether “average” results can apply to single patients [2]. Over the past decades, two key advances have changed the landscape of cancer research: a predominantly reductionist approach in biological research has given way to the widespread use of omics and high-throughput techniques [3,4,5,6], while the therapeutic armamentarium, previously based on chemotherapy and a few hormonal drugs, has been enriched with a large variety of targeted drugs [7]. The complexity [8,9] and heterogeneity [10,11] of tumors has prompted the development of precision oncology, aiming to tailor treatments to individual patients [12,13,14,15,16,17]. Methods have been developed to deal with the heterogeneity of treatment effects in RCT [18,19,20], and new clinical trial designs have been proposed, often adopting Bayesian methods and adaptive principles, to deal with the need to develop treatments based on tumor biological features [21,22,23,24].

Nonetheless, there remains a gap between the current possibilities to characterize the entire spectrum of molecular alterations of a single tumor and our capacity to define the best treatment based on those molecular alterations. In this paper, we will review current systems biology methods and provide examples of how they can be valuable tools to fill this gap and further the development of cancer treatment. We avoid most technicalities and primarily target an audience of medical oncologists.

## 2. Precision Oncology Challenges

Precision oncology emerged as a new paradigm with the advent of drugs whose molecular targets are precisely defined. The targeted activity depends on the status of the target itself, often constitutively active [25,26]. Many new drugs target intracellular signaling pathways, but others target processes occurring in the microenvironment, such as neo-angiogenesis, or the immune system. The fundamental intent of precision oncology is to match treatments to the molecular alterations that are present in the individual tumor to increase the chances of efficacy.

Precision oncology requires a deep biological characterization of tumors, usually performed through omics studies. This has led to the identification of tumor subtypes within various primary tumors [27,28], useful to stratify patients for prognostic and therapeutic purposes. More detailed classifiers have been derived from the joint analysis of different types of omics data [29,30,31,32,33].

A key aspect of precision oncology is predicting the individual response to treatments. The presence of the target, often represented by a mutant (e.g., kinase-activating mutation in EGFR, BRAFV600E), overexpressed (e.g., HER2), or fusion protein (e.g., BCR/ABL and NTRK), is often the prerequisite for the use of a molecularly targeted drug [34,35]. Identification of the target, by gene or protein expression analyses, is therefore commonly sufficient to set the indication for the use of a targeted drug in clinical practice [36,37]. However, the predictivity of such single biomarkers is limited, and predictors formed by panels of biomarkers are being widely studied.

Transcriptomic assays are used to predict the benefit of adjuvant chemotherapy in patients with hormone receptor-positive, human epidermal growth factor receptor 2 (HER2)-negative breast cancer [38,39,40]. They have also been applied to predict the benefit of radiation therapy [41].

Transcriptomic predictor panels could be even more relevant for therapies targeting intracellular signaling pathways, as exemplified by the HER2 pathway in breast cancer. The PAM50 transcriptomic predictor has been shown to predict response to HER2-targeted therapies better than the standard evaluation of HER2 overexpression or gene amplification [42]. In genomic alteration studies, considering groups of alterations affecting the same pathway yields a better prediction of response to anti-HER2 therapies than mutations in single genes [43]. The measurement of the total and phosphorylated levels of signaling molecules could help to further refine predictions [44,45], while integrating phosphoproteomics with genomics and transcriptomics analyses can increase the capacity to identify driver molecular alterations and therapeutic targets [46,47,48].

Similar efforts have recently been undertaken to predict the response to immune checkpoint inhibitors (ICIs). The tumor or immune cell expression of Programmed cell Death-Ligand 1 (PD-L1), assessed by immunohistochemistry, is a standard method to select patients for treatment with ICIs, but has limited predictivity and standardization [49,50]. Mismatch repair deficiency and a high tumor mutational burden are tumor “agnostic” markers that qualify patients for treatment with pembrolizumab [51]. The acknowledgment of the complex, multifactorial nature of the interactions between tumor and the immune system has suggested the introduction of the “cancer immunogram” as a collection of multiple biomarkers to guide treatment choice [52]. Gene expression signatures have been studied as predictors of response to ICIs (e.g., an interferon-γ signaling signature) [53], or as predictors of resistance [54]. Although most of these panels have not entered clinical practice so far, these examples show that predictors based on panels of biomarkers can outperform single biomarkers.

Another key aspect of precision oncology is deciphering the mechanisms of resistance to targeted drugs and identifying strategies to overcome them. Resistance may be due to alterations in the target itself, which is no longer present (e.g., after the emergence of HER2-negative clones in a tumor originally HER2-positive) [55] or undergoes secondary mutations that make it resistant to inhibition by the drug (e.g., EGFRT790M in non-small cell lung cancer (NSCLC)) [56]. Sometimes mutations arise in molecules downstream of the drug target (e.g., mutations in RAS, RAF, and PI3K during EGFR inhibitor therapy) [56,57], leading to the constitutive activation of a pathway. However, drug resistance may also depend on network adaptation mechanisms, including facilitation of resistance by feedback mechanisms (e.g., multiple feedbacks present in the MAPK pathway) [58], or activation of parallel pathways that bypass inhibition (e.g., amplification of MET or HER2 in NSCLC treated with EGFR inhibitors; and crosstalk between estrogen receptor and HER2 pathways in breast cancer) [56,57,59].

A further critical goal of precision oncology is to identify optimal drug combinations that can increase therapeutic efficacy and overcome drug resistance. The dual targeting of a receptor with two drugs acting with different mechanisms (e.g., trastuzumab in combination with pertuzumab or lapatinib as anti-HER2 drugs) has led to improved efficacy in RCTs [60,61]. The same occurred with the combination of two drugs acting on different molecules along the same pathway, e.g., a BRAF inhibitor and a MEK inhibitor [62,63,64]. 

The complexity of intracellular signaling networks and cell-cell interactions among the various systems in the tumor microenvironment makes it particularly challenging to identify the interactions responsible for drug resistance and design strategies to overcome it. The various aspects considered above suggest that a systems approach is essential in precision oncology.

## 3. Systems Biology

Systems biology studies the collective behavior of different types of molecules involved in a biological process, aiming to reconstruct the system behavior. Systems of many different molecules have behaviors that cannot be simply deduced from the properties of their constitutive elements, requiring a higher level of analysis to be understood, pertaining to systems theory [65,66,67]. Biological entities are dynamical systems that evolve in space and time. A snapshot of the system at a certain time point, showing the spatial disposition and the concentrations and activities of molecules, is called a state. As intrinsic noise and environmental perturbations inevitably occur over time, the system evolves passing through different states. The sequence of the states, describing the evolution of the system, can be represented graphically as a trajectory in the so-called state space. The aim of systems analysis is to describe the evolution of the system, e.g., following the occurrence of mutation or a drug treatment. Knowing the state of the system at a certain initial time, this analysis aims to predict the state of the system at a future time and the contributions of different constituents into the control of system evolution. 

Biological systems have usually a network configuration, resulting from the interaction of smaller functional modules, also called motifs, each characterized by some specific dynamic behavior. The connections between modules quantify how a module affects another module and include feedback and feedforward loops [68]. The interplay of these modules leads to the acquisition of properties that characterize complex systems, called “emergent properties” [69]. 

Dynamical systems evolve towards specific states, called attractors. These may be a fixed, steady state, but also a closed trajectory in the state space, called limit cycle, and presenting oscillating behavior. There may also be more complicated trajectories in the state space that have completely irregular shapes, called strange attractors. These characterize chaotic dynamics, resembling a completely irregular stochastic behavior, although determined by deterministic non-linear rules [70]. A system can have more than one attractor, e.g., two different stable steady states (a condition called bistability) and evolve toward one or the other depending on the starting state of the system. The behavior of a dynamical system depends on the value of parameters (such as the rate constants of enzymatic reactions); as these vary, the behavior changes in a quantitative or qualitative way. The condition, in which the system dynamics undergoes an abrupt qualitative change, when one or more parameters cross some critical values, is called “bifurcation”. For example, a system that tended to converge towards one of two steady states, can begin to oscillate between the two. 

### Cancers as Dynamical Systems

The typical emergent properties of systems are their robustness, which defines the ability of a system to keep its function despite external or internal perturbations, and adaptation, referring to the ability of a system to adjust its behavior in response to environmental changes. Robustness and adaptation characterize tumors as complex systems, which almost inevitably adapt to anticancer drugs and develop resistance [71,72]. Ways to overcome resistance are among major goals and promises of precision clinical oncology.

An array of statistical and mathematical modeling techniques can be applied to describe, with different levels of accuracy, dynamical biological processes. We will illustrate some approaches, referring the reader to recent excellent reviews for more comprehensive descriptions [73,74,75,76,77,78,79,80]. 

## 4. Statistical Methods

Statistical models aim to find associations among variables and are commonly applied to analyze medical and biological datasets. Supervised statistical methods deal with different predefined classes of objects (e.g., responders versus non-responders) and try to identify a set of variable values that help us discriminate among classes. Unsupervised methods consider the whole set of data without prior classification and aim to identify relevant inherent subsets in the data. Supervised methods include the different types of regression models: linear, logistic, Cox proportional hazards regression, etc. [81]. To enable the analysis of omics data, in which the number of variables is much larger than the number of samples, the so-called robust regressions are commonly adopted [82]. Unsupervised methods include cluster analysis [83], aiming to identify relevant inherent subsets (clusters) in a dataset, and principal component analysis [84], pointing to reduce the number of variables (dimensionality reduction) by combining original variables into a few new, condensed variables (principal components) with minimal loss of information. Partial least squares regression makes the same dimensionality reduction, but in a supervised context [84,85].

The statistical methods applied to omics data typically yield long, ranked lists of variables that are significantly associated with a phenotype or response variable. A useful tool is gene set analysis [86] that considers groups of related genes, defined by their involvement in a specific cellular process, function, or pathway. It then assesses the association of each gene set to the phenotype of interest, pointing out the processes or pathways that subtend the phenotype. 

In addition to identifying tumor subtypes and differences between groups, as highlighted in the section on precision oncology, omics studies have been used to identify genes and pathways of clinical relevance. In many patients, cancer cells develop resistance to ionizing radiation (IR). Recently, an integrative bioinformatics analysis was applied to publicly available transcriptomic datasets of human cancer cells of different tissue origins treated with IR [87]. Supplemented with literature data, this analysis led to the identification of a panel of interconnected genes, belonging to pathways involved in neoplastic processes and implicated in the mechanisms of resistance to IR. Transcriptome profiles from microdissected cancer-associated fibroblasts and ovarian cancer cells have been analyzed with a computational model to decipher the stroma-cancer crosstalk based on ligand-receptor interactions and downstream signaling networks. This analysis identified Smad signaling as a poor prognostic factor in a cohort of ovarian cancer patients, while Smad inhibition by calcitriol, found through a drug repositioning program, inhibited tumor progression in ovarian tumor bearing mice [88].

A limitation of omics studies is that only a few papers report data of cell responses to systematic perturbations, although such data appear to be the most informative datasets [89,90,91]. Merely identifying an association between variables does not imply the existence of a causal relationship. Future analyses will require perturbation omics data, and the term perturbation biology was recently coined [92]. 

Machine learning is the part of artificial intelligence (AI) that uses computer algorithms to analyze big datasets to generate predictive models. These algorithms employ statistical tools, both supervised and unsupervised, and are capable to iteratively self-adjust to optimize the performance [93]. They require big training sets and cannot provide predictions on drugs or targets that were not included in the training set. The AI tools have recently been discussed in several excellent reviews [93,94,95,96]. 

## 5. Network Analysis

**Figure 1:**
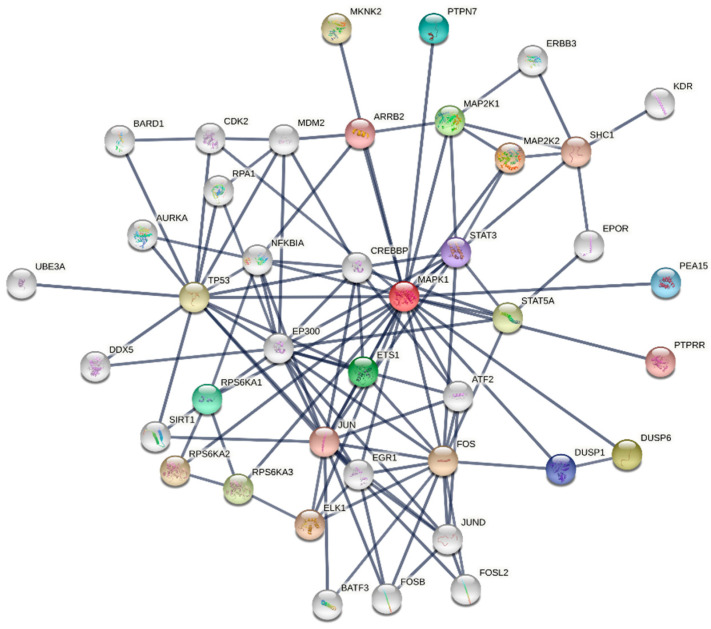
Interaction network of MAPK1. The network is built using the STRING database [100]. The top 20 first neighbors and top 20 second neighbors of MAPK1 (aka ERK1) are shown. The thickness of the edges is proportional to the confidence of the edges and the highest interaction score of 0.90 is selected to remove low-confidence connections.

Another tool to decipher high throughput data is constructing networks of interconnected molecules, followed by the network analysis [97,98]. Different types of network representation exist [99], including graphs where the variables and their interactions are symbolized by circles, called “nodes”, and lines, called “edges”, connecting the nodes (Figure 1). The connections may involve a physical interaction or a functional linkage between two variables. The edges may be undirected, showing only connections between nodes, whereas directed edges specify a direction of the relationship from a source node to a target node. In the field of proteomics, for instance, the first are used to reconstruct the occurrences of physical binding among proteins in the so called “protein interaction networks” or “interactomes”, and the latter to reconstruct the flow of protein phosphorylation events in “protein signaling networks” [98]. Directed edges may be activating or inhibitory. 

Network analysis aims first to define the structure or topology of a network. Topology refers to a set of geometrical properties (those not affected by continuous deformations) of a network and is linked to the functional properties. Nodes are characterized by various attributes, such as the degree, which represents the number of edges attached to a node. Based on nodes features, some network features can be defined, such as their average degree and their degree distribution, representing the percentages of nodes for each degree. Most biological networks show many low-degree nodes and a few high-degree nodes called hubs. These types of networks, also called scale-free networks, are contraposed to the so-called random networks, where the degree distribution is more concentrated around a mean. General features of scale-free networks are their robustness against accidental failures due to random nodal damage, and vulnerability to targeted attacks against hub nodes [98]. However, this generic network description provides limited insights into directionality of signal flow, the regulation and functions calling for a dynamic quantitative network description. 

A major task of systems biology is to quantify and reconstruct the dynamic connections between genes, as well as proteins, which determine network responses. Biological networks can be reconstructed following a “bottom-up” (or knowledge-based) approach, whereby the selection of molecules and interactions to be included in the network is based on information extracted from the literature and public databases. Alternatively, a top-down (or data-driven) approach can be followed, reconstructing the network directly from experimental data, for example from omics studies [99]. 

Data-driven network analysis attempts to reconstruct intracellular networks from high throughput data, a task called the “reverse engineering” [101,102]. High throughput data are collected under different cellular states, e.g., before and after some perturbation, yielding several snapshots of the molecular profile of the cell population in different conditions. Then the interactions among the measured molecules are reconstructed, by assessing hints, such as which molecules vary synchronously or in temporal sequences. Different tools are used to reconstruct the interactions among molecules. Some are statistical tools based on correlation or regression [103]. A typical tool are Bayesian networks [104]. The Bayes theorem allows to estimate the probability of an event A conditional to B (that is, given a certain value of the event B) starting from the probability of B conditional to A (inverse probability). Bayesian networks allow to estimate the probability of a certain set of molecular interactions from the different snapshots of molecular profiles given the experimental data. The Bayesian formalism is also used to estimate parameters and derive models from experimental data [105]. Bayesian networks can combine variables of different nature, but cannot infer feedback loops [101]. Dynamic Bayesian networks represent an extension of Bayesian networks to model the temporal evolution of a dynamical system, inferred from time-course data [106,107]. By representing each variable multiple times, one for each time point considered, these models can reproduce the activation of sets of nodes and/or edges along time, including cyclic regulatory relationships such as feedbacks [107].

Another reverse engineering approach applies concepts from information theory, such as mutual information, a measure of relatedness, to identify sets of coregulated genes, and the “data processing inequality”, to eliminate indirect relationships among genes and to highlight direct interactions. Contrary to methods based on statistical correlation, it can capture also non-linear dependencies among nodes. Compared to Bayesian networks, this method proved superior in reconstructing the interactions of a synthetic genetic network model, and was successfully used, for instance, to reverse engineer the regulatory networks of human B lymphocytes [108]. From networks, it is possible to identify “master regulators”, key molecules affecting specific disease-related phenotypes [109].

Reverse engineering can also be tackled by dynamical system-based approaches, such as modular response analysis (MRA), which addresses this task by dividing a global signaling network into several smaller functional units or modules and focusing on intermodular interactions. MRA quantifies network connections in terms of a response of a single module to a perturbation of another module that directly affect that module, assuming that all other modules are fixed to prevent the propagation of this perturbation through the entire network. By making perturbations to each module and measuring the global network responses, it is possible to retrieve the connections between single modules, including feedback loops, thereby quantitatively reconstructing the interaction map, including feedback loops [110,111]. A recent benchmarking of reverse engineering methods found that MRA performance surpassed correlation and mutual information-based methods [112]. MRA can be combined with a Bayesian variable selection algorithm to account for biological noise and measurement errors. When used to infer the ERBB and G1/S transition pathways in breast cancer cell lines, it successfully identified many known pathway interactions as well as feedback interactions promoting trastuzumab resistance [113]. This Bayesian implementation of MRA (termed BMRA) has been further developed to improve the inference precision [114]. In contrast with classic Bayesian network methods, BMRA reconstructs the normalized Jacobian (the matrix of all first-order partial derivatives) of a dynamical system, commonly described by ordinary differential equations (ODEs). Therefore, BMRA allows to build a mechanistic, dynamic ODE model based on the inferred quantitative topology of a network. BMRA can reconstruct the network circuitry using much smaller datasets than Bayesian network methods, because the BMRA likelihood function is based on the deterministic equations of MRA. In addition, BMRA requires fewer perturbations than deterministic MRA, is tolerant to noise, and allows to incorporate existing pathway knowledge as a prior network to improve the inference precision [115]. Even when this knowledge is inaccurate for half of the network edges, BMRA recovers a nearly perfect network topology as validated in independent experiments [114]. Based on BMRA reconstruction of EGFR and insulin-like growth factor 1 receptor (IGF1R) pathways, ODE models were built for six different colorectal cancer cell lines [114]. These models highlighted cell line specific network rewiring and identified a negative feedback from p70S6K to insulin receptor substrate 1 (IRS1) as responsible of resistance to EGFR inhibition in some cell lines. Model simulations suggested that disrupting this feedback may restore sensitivity to EGFR inhibitors, a prediction that has been experimentally validated in cell cultures and in a zebrafish xenograft model [114]. 

## 6. Logic Models

Logic models represent the interaction between two molecules in a system as a logical statement, spanning among three levels of accuracy: binary (Boolean) logic, multi-state, and fuzzy logic [78,99,116]. In Boolean logic, each variable (molecular species) can be in one of two possible states (true or false, on or off, 1 or 0). A Boolean network is formed by a set of Boolean variables, representing the nodes of the network, and the connections among variables are defined by Boolean functions. The latter are represented, in the so-called “truth tables”, by couples of attributes, specifying the states of the two variables. An activating signal may be represented as 1/1, meaning that when the first node is activated (1, on), also the second becomes activated. An inhibitory signal may be represented as 1/0, meaning that when the first node is activated, the second is inhibited (0, off). Logic modeling does not require knowledge of the detailed mechanistic relationship between nodes, but simply represents the direction and the type (e.g., activating or inhibitory) of relationship. By combining more couples of attributes, it is possible to specify the logic operators AND (when two activated upstream nodes are needed to activate the downstream node), OR (when the activation of any of two upstream nodes is sufficient to activate the downstream node), and other logical gates [117]. The state of a variable therefore depends on the combination of the logical relationships in which it is involved. This modeling approach, despite its oversimplification, can reproduce the functioning of biological networks. Increased accurateness, at the expense of computational cost and complexity, can be acquired by considering multi-state discrete models, which specify additional discrete levels between 0 and 1 (corresponding, for instance, to no, low, and high activity of a molecule), and by fuzzy logic models, which consider the infinite spectrum of real numbers between 0 and 1 as possible truth values of a variable. Although logic models mainly yield qualitative results, they can reproduce the evolution of a system, highlighting the attractors toward which the system tends to evolve. They are suitable to predict the effects of perturbations, such as mutations or exposure to drugs, on a system’s behavior. 

Logic models were used to analyze the immediate-early responses of human hepatocellular carcinoma cells to seven cytokines and growth factors, in presence or absence of seven small-molecule kinase inhibitors [118]. Starting from a literature-derived network of 82 proteins mediating this response, they measured the abundance and phosphorylation state of 16 molecules, before and 30 min after the exposure to each cytokine or growth factor, with or without an inhibitor. After reducing this system into a “compressed” network with 31 nodes and 53 edges, the authors considered all the possible Boolean models, selecting the model with the least possible number of variables, which more closely simulated the experimental data. The final model turned out to include considerably fewer connections than the “compressed” network, dismissing the connections not relevant in that cellular context. It also added a few connections, not present in the original network, that increased model data fitting, and that turned out to have literature support. To validate the model, hepatocellular carcinoma cells were exposed to a combination of two cytokines or growth factors, in the presence or absence of one of four kinase inhibitors. The model fitted this validation set of data nearly as well as the training set from the previous experiment, showing a good predictive power in terms of false-negative and false-positive results. Nevertheless, logic network modeling has several limitations. The exponential increase in the number of states with the number of logical network nodes makes the number of attractors computationally intractable and hinder biological interpretation of computed network responses [119].

## 7. Mechanistic Models

Mechanistic models that are based on systems of differential equations require a more detailed knowledge of the molecule structures and biochemical reactions involved. Building a mathematical model of a biological process requires (i) establishing which molecules and interactions to consider, (ii) choosing mathematical expressions to describe each interaction, (iii) finding suitable values for the parameters involved, and then (iv) solving the equations to simulate the behavior of the system, thereby making predictions about system responses to a perturbation of one or more components [65,99,120,121,122]. 

The simplest form of differential equations are ODEs. These models use the mean field approximation and can accurately describe the time evolution of a biochemical systems if the number of molecules of each species is large enough [123]. ODEs relate a function (or several functions) with its (their) derivative. The concentration or activity of a molecule is a function of time, and its derivative represents the rate of change of this concentration. ODEs are therefore particularly suitable to study the dynamics of a biological system, describing its evolution over time. Modeling such systems with ODEs, typically involves considering all the processes that modify the level or activity of each relevant molecule, such as synthesis and degradation, import, and export from a cell or compartment, activation or inhibition (e.g., by phosphorylation or dephosphorylation), binding or dissociation, while taking into account the stoichiometries (the quantitative relationships among the substances that participate in a chemical reaction) of each reaction. These processes are characterized by rate parameters, such as kinetic constants, appearing in the ODEs, and whose values must be measured or estimated experimentally. Solutions to ODEs are functions of time, which are therefore represented not as single values, but as plots on Cartesian axes, depicting the evolution of the state of the system over time. Beyond depending on the values attributed to the parameters of the model, the solutions depend also on the so-called “initial conditions”, such as the initial concentrations. 

The biochemical reactions can be modeled according to the laws of chemical kinetics [120,124]. The most basic reactions, called also elementary reactions, follow the law of mass action, whereby the rate of the reaction is proportional to the concentrations of the reagents. This reaction rate is equal to the rate constant multiplied by the product of the concentration of the reactants (with possible exponents depending on the stoichiometry and order of the reaction). From the elementary reactions, the kinetic laws of more complicated reactions can be derived, such as the Michaelis-Menten enzyme kinetics, which assumes a quasi-steady state, or rapid equilibrium of the enzyme-substrate complex [125]. When the biochemical kinetics of reactions involved are not known in detail, the relationship between two interacting molecules can be approximated by means of generic functions, such as the Hill function, commonly used to model the receptor activity as a function of the ligand or drug concentration. This is a sigmoidal function, one of whose parameters, called the Hill coefficient, is responsible for the steepness of the curve. If the Hill coefficient is 1, there is the Michaelis-Menten relationship between the ligand concentration and the activity of the receptor, whereas for increasing values of the Hill coefficient, the curve becomes more step-like, simulating the existence of a threshold. 

If the assumption of high molecule numbers (more than 1000) are not fulfilled, stochastic models must be used, such as the chemical master equations (CME), which, instead of considering only numerical values of variables, consider a probability distribution for each variable [126,127,128,129]. These models, based on stochastic differential equations, allow to take into account stochasticity and heterogeneity inherent into biological processes and random errors in their measurements, at the cost of the increase in computational effort. While analytical solutions can be found only for very simple CMEs, these can be solved by common numerical methods. More frequently, as CMEs represent probability distributions, they are analyzed by the so-called stochastic simulation algorithms (SSAs), such as the Gillespie algorithm and several of its approximations [130,131]. 

A precise modeling of chemical reactions requires to represent each different state of any single molecule (e.g., unphosphorylated, or phosphorylated at one site, or at two sites, etc., or unbound, or bound to one or more molecules in a complex, etc.) as an individual variable. This leads to an exponential increase in the number of variables, and of the corresponding ODEs, involved in each reaction, to account for all possible combinations of molecular states and relationships. To overcome this combinatorial explosion of variables, the approach of rule-based modeling has been developed. Each rule determines a set of chemical reactions, whose rates depend on the conformational, phosphorylation and spatial localization states of molecules [132]. These rules are directly transformed into systems of ODEs by specific software packages, such as BioNetGen [133,134]. Models that are built in BioNetGen can be integrated by the Network-Free Stochastic Simulator (NFsim) [135,136]. NFsim can use the Gillespie SSA and consider noise, intrinsically present in biological systems, for instance, in transcription factor networks [78]. 

## 8. Emerging Network Properties Captured by Differential Equation Models

Differential equations are widely used to model complex biological networks that mediate responses to intracellular and extracellular inputs, activating cellular functions, such as proliferation, differentiation, senescence, and apoptosis [122,137]. The derangement of these networks, due to genetic or epigenetic alterations, is at the basis of cancer development and progression [8,9,138]. Cellular receptors share a common downstream network, and the specificity of the output depends not only on the ligand-receptor pair, but also on subtle differences in the temporal and spatial dynamics of signal transduction throughout the network, including different signal features, such as amplitude, duration, frequency and spatial distribution [67]. For instance, epidermal growth factor induces a transient extracellular signal-regulated kinase (ERK) activation in MCF-7 breast cancer cells, eliciting proliferation, whereas heregulin induces sustained ERK activation causing differentiation [139]. 

**Figure 2:**
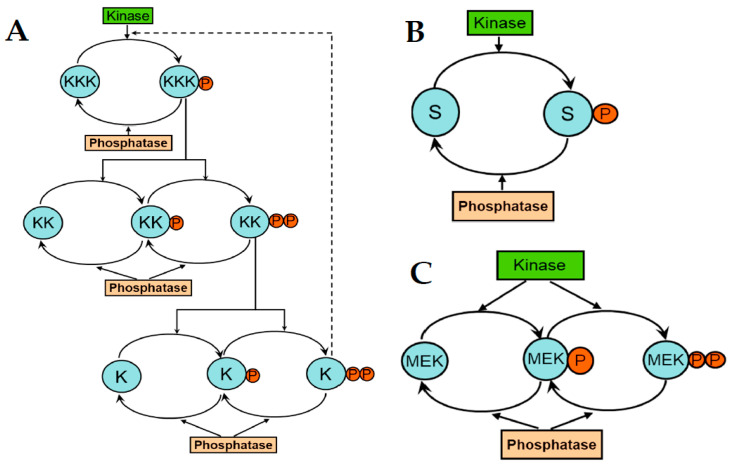
A cascade of protein (de)phosphorylation cycles. (**A**) The 3-tier cascade has a similar structure to MAPK cascades (KKK/KK/K). The phosphorylation of two sites is necessary for full activation of the kinases KK and K. Feedback loop is shown by a dashed line. (**B,C**) Typical cascade motifs are mono- and double (de)phosphorylation cycles.

Signal transduction in most cellular networks occurs via cascades of protein (de)phosphorylation cycles (Figure 2A). A typical cycling motif is formed by two or more states of a protein, phosphorylated and dephosphorylated, which are controlled by opposing enzymes, a protein kinase and a phosphatase (Figure 2B,C). Even mono-phosphorylation cycles show peculiar behaviors, such as ultrasensitivity, whereby, when the converting enzymes operate near saturation, the response to an input becomes abrupt, and is represented by an extremely steep sigmoidal curve, instead of the more common hyperbolic curve describing enzyme kinetics [140]. Ultrasensitivity increases as the number of interconnected cycles in a signaling cascade increases [68], and is further potentiated in multi-site phosphorylated proteins (Figure 2C), which can produce switch-like responses [141]. 

**Figure 3:**
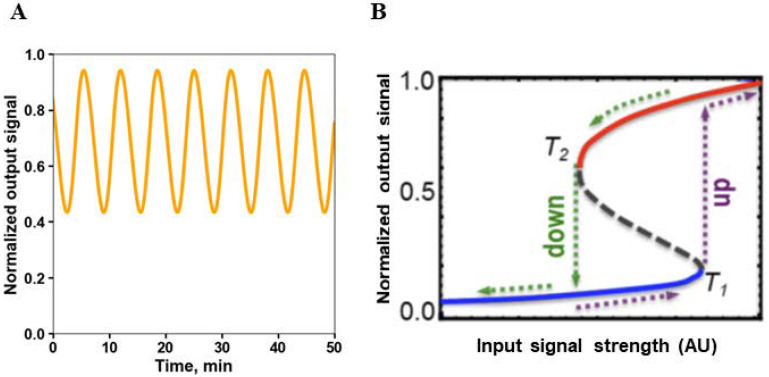
Oscillations and hysteresis brought about by negative and positive feedback loops. (**A**) Typical sinusoidal shapes of oscillations caused by strong negative feedback. (**B**) Hysteresis, a hallmark of bistability, is often triggered by positive feedback. When the input signal increases over the threshold ($T_{1}$), the output pathway activity, which initially gradually increased with the input signal, abruptly jumps to a much greater value and then again gradually increases with the further signal increase (the direction of the changes is shown by dashed brown arrows). Following this jump to the higher activity state, the output activity remains high with the decrease in the input signal, and only when this signal reduces to the value ($T_{2}$) that is much smaller than $T_{1}$, the output activity returns to the low activity state (dashed green arrows).

Another fundamental feature of networks are feedback loops. Positive feedbacks amplify the signal, whereas negative feedbacks attenuate it, facilitating system’s adaptation and robustness to noise [142]. However, feedbacks can also favor the occurrence of instabilities, leading to a radical change in the state of the system. Too strong, long negative feedbacks induce damped or sustained oscillations, usually with a sinusoidal shape [143] (Figure 3A). Positive feedbacks can cause bistability and hysteresis, where the threshold for jumping from one steady state to the other differs depending on the direction of change of an external signal or parameter (Figure 3B). Hysteresis has been found in experiments on cell cycle control in *Xenopus* oocytes. Progressively adding cyclin leads to gradual activation of cyclin-dependent kinase 1 (CDK1), until when, beyond a certain threshold (C_high_), activation of CDK1 jumps to remarkably higher levels. When cyclin levels are progressively reduced, CDK1 activation diminishes gradually, but only when cyclin levels diminish below a threshold C_low_, much lower than C_high_, does CDK1 activation drop to its original level [144,145]. Therefore, hysteresis prevents the easy reversal of a system state, committing to its sustainability. Positive feedbacks occurring in combination with negative feedbacks can give rise to sustained oscillations, called relaxation oscillations, typically observed in cell cycle regulation. They are characterized by a pulsatory shape, producing alternating “off” and “on” states of the system. The presence of bistability/hysteresis and relaxation oscillations driving cell cycle had been suggested by mathematical models of the cell cycle long before being experimentally observed [146]. 

Under some conditions, hysteresis gives way to an irreversible switch to one of the two steady states, marking the commitment to a cellular fate, thus converting a transient stimulus in an irreversible response. Maturation of oocytes in *Xenopus*, in response to a short exposure to progesterone, is determined by positive feedbacks within the p42 mitogen-activated protein kinase (MAPK)/cell-division cycle protein kinase Cdc2 pathway [147]. Hysteresis and irreversible switches allow the control of multiple irreversible transitions in cellular processes, such as those occurring at cell cycle checkpoints. Using single cell analyses and mathematical modeling, it has been shown, for instance, that the Retinoblastoma-E2F pathway functions as a bistable switch, converting graded mitogenic stimuli into an all-or-none proliferative response [148].

Whereas ODE models precisely describe the time evolution of the mean concentration and activity values, external and internal noise intrinsic, for instance, for transcription can change the systems dynamics [149,150]. If a dynamical system state is in the vicinity of the border between two basins of attraction, noise can flip the system to a new attractor, for instance, to a new steady state of a multi-stable system. In this case, fluctuations are no longer small corrections to the time evolution of the mean values, but a key event that causes switching of cell states. The introduction of stochastic terms into equations describing the behavior of multi-stable systems has led to a quantitative interpretation of Waddington’s epigenetic landscape in terms of nonlinear stochastic dynamics [151,152]. The original and now prominent Waddington’s model viewed cells moving through a landscape of mountains and valleys as rolling marbles from one state to another. In recent stochastic approaches, Waddington’s landscape is treated as a landscape of the probabilities for the system states in the presence of noise, while the local minima of this landscape are the steady states determined by ODEs. This view allows determining the rate of transitions between different basins of attraction, that is, different cell states, based on the circuitry of the network governing cell fate decision [153,154].

## 9. Modeling Spatiotemporal Network Behavior by Partial Differential Equations

The use of ODE models requires the fulfillment of the assumptions that the molecules are evenly distributed in the modeled compartment, and this condition is not fulfilled for molecules bound to membranes or molecular scaffolds. Often, opposing enzymes, such as a kinase and phosphatase in signal transduction cascades, are localized to different cellular compartments (e.g., a kinase resides at the cell membrane, whereas a phosphatase is homogeneously distributed in the cytosol). This results in the emergence of gradients of phosphorylated and unphosphorylated forms of the protein substrate [155]. Because in this and similar cases, the variables (concentrations) depend not only on time, but also on the spatial coordinates, describing the rate of change of these concentrations requires so called “partial differential equations” (PDEs), introducing a further level of complexity. PDEs describe the spatiotemporal behavior of species and are derived by coupling the ODE dynamics with species diffusion and membrane transport [156]. Recently, the minimal autonomous biochemical machinery of RhoA GTPase necessary and sufficient to govern cell movement was established using experiments and PDE modeling [157]. Ras homolog family member A (RhoA) controls the contractility at the cell rear, whereas Ras-related C3 botulinum toxin substrate 1 (Rac1) controls protrusions and retractions at the leading edge [158,159]. In the cell rear and body, RhoA and Rac1 mutually inhibit each other, but at the leading edge of a migrating cell, RhoA activates Rac1 via the effector, diaphanous-related formin-1 (DIA1) [160]. In the ODE model, this combination of negative and positive feedforward and feedback loops lead to bistability and oscillations but coupled with species diffusion in the PDE model these dynamics result in periodic, propagating waves of RhoA and Rac1 activities that control cell migration [157]. When a periodic wave that starts at the leading edge reaches the cell rear, it induces transient RhoA-Rac1 oscillations, RhoA activity spikes and retraction of the rear. After the rear retracts, the initial GTPase network dynamic pattern resumes. These PDE model predictions were fully confirmed by live cell imaging. Thus, PDE modeling, combined with experiments, suggested a new concept in cell migration research by showing that distinct GTPase dynamics at the cell leading and trailing edges reported previously are coordinated by periodic, propagating RhoA-Rac1 waves.

## 10. Mechanistic Models Help Us Understand Resistance to Targeted Therapies

Cellular network adaptations are involved in the development of resistance to targeted therapies. Resistance occurs due to reactivation of the same signaling pathway or the activation of alternative pathways. To better understand resistance, signaling networks, including receptor tyrosine kinases and their downstream MAPK and PI3K/AKT/mTOR pathways that are often associated with resistance, have been intensively modeled [67,161,162,163,164]. 

A classic example is resistance to BRAF inhibitors in patients with melanoma, characterized by the paradoxical activation of the MAPK pathway due to increased formation of RAF homo- and hetero-dimers, particularly BRAF-CRAF heterodimers [165,166]. RAF dimerization is a key step in the physiological activation of RAF kinases, dramatically increasing their catalytic activity [167]. It is induced by binding of RAF kinases to RAS, but is also increased by treatments with RAF inhibitors, which induce conformational changes in RAF molecules leading to increased dimerization affinity. If only one RAF protomer is inhibited in a RAF-dimer, the inhibited protomer allosterically activates the other RAF protomer. A quantitative model suggests that upon RAF dimerization, for thermodynamic reasons, the affinity of a BRAF inhibitor for one of the RAF protomers increases, but the affinity for the other protomer sharply decreases. This favors the formation of RAF dimers where only one protomer is bound to the inhibitor, with consequent allosteric activation of the inhibitor-free protomer and sustained MAPK signaling activity [168]. 

ATP-competitive inhibitors can be classified into type I, I½, and II based on their preferential binding for one of the kinase conformations. The same model suggests that a combination of two structurally different inhibitors, binding to different RAF conformations, can overcome resistance, also when the two inhibitors are ineffective on their own, and even if given at doses lower than those used in monotherapy. To confirm this prediction, a more comprehensive mechanistic, rule-based ERK pathway model has been developed, which integrates the structural, thermodynamic, and kinetic analyses of RAF (BRAF and CRAF) kinases-RAF inhibitors interactions, their interactions with other molecules of the pathway (RAS, MEK, and ERK), as well as cellular genetic profiles (RAS and RAF mutation status) [132]. The model’s predictions were compared with experimental interventions on melanoma cell lines. The model faithfully predicted RAF inhibitor responses in BRAF-mutant, RAS wild-type cells, as well as in RAS-mutant cell lines, confirming the effectiveness of a combination of two structurally different RAF inhibitors, such as vemurafenib plus sorafenib, in inhibiting ERK signaling, cell proliferation and colony formation, even in RAS-mutant cell lines. This paradoxical pathway activation can be facilitated by negative feedback loops, when the alleviation of the feedback induced by a kinase inhibitor leads to further increase in kinase dimerization [169]. The model allows to estimate the levels of synergy or antagonism between RAF inhibitors and MEK inhibitors, based on the type of RAF inhibitor and the level of RAS activity. In cells with low RAS activity, such as wild-type RAS, BRAFV600E melanoma cells, there is synergy between type I½ RAF inhibitors (e.g., dabrafenib or encorafenib) and MEK inhibitors (trametinib or binimetinib). On the contrary, in cells with high RAS activity, such as those with mutant RAS, this combination may turn antagonistic at low drug concentrations, or even at high drug concentrations for vemurafenib, that has a wide dose range of paradoxical activation. Type II RAF inhibitors, such as TAK632, have a narrower range of paradoxical activation, but antagonism with MEK inhibitors is still present at low inhibitor doses and higher doses are required for synergy. A combination of types I½ and II RAF inhibitors reduces the dose ranges of paradoxical activation, yielding synergy over a wider dose range than a combination of RAF and MEK inhibitors in RAS-mutant cells. Experiments conducted on RAS-mutant melanoma and acute myeloid leukemia cell lines support these model predictions [169].

**Figure 4:**
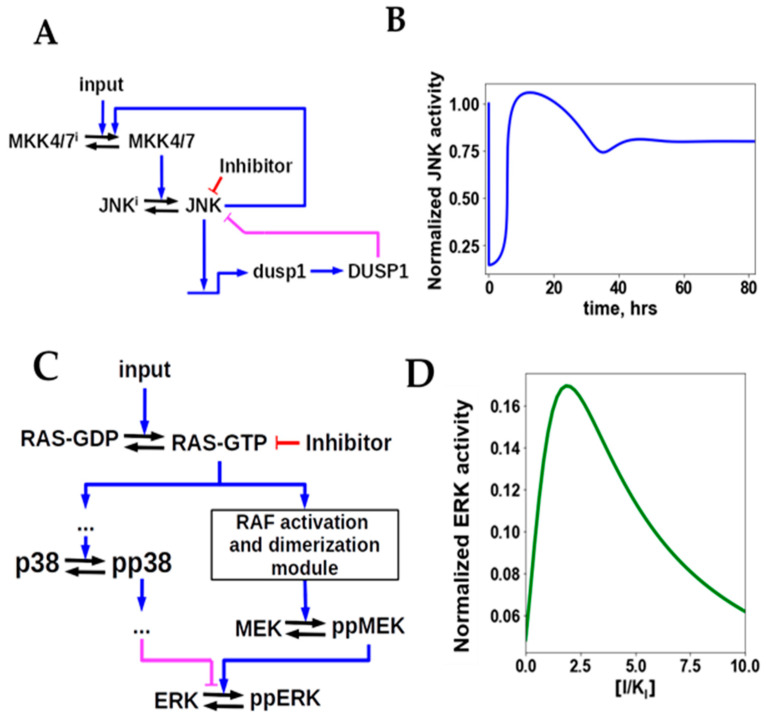
Feedback loops cannot completely reactivate steady-state output signaling and network adaptations that can fully restore or increase it. (**A**) A simplified diagram of the JNK/MAPK pathway featuring negative transcriptional feedback via the induced phosphatase (DUSP) synthesis and positive phosphorylation-mediated feedback loops. (**B**) The time course of the JNK activity after the JNK inhibitor treatment. A transient reactivation period and overshoot is followed by the decrease in the steady-state JNK activity. (**C**,**D**) Two feedforward connections, positive and negative, can lead to complete signaling reactivation. (**C**) Positive and negative feedforward routes from RAS to ERK mediated by RAF/MEK and the p38 pathway, respectively. (**D**) Dose response curve shows paradoxical activation of ERK by inhibitor (I) within a certain dose range.

Negative feedbacks have been held responsible for the development of acquired resistance to many targeted drugs [170,171,172,173,174,175]. This hypothesis was addressed by a systematic analysis of network adaptation mechanisms as a cause of drug resistance [169]. The unbiased analysis and mechanistic modeling demonstrated that feedback loops, by themselves, could not completely reactivate steady state signaling. Following drug inhibition, negative feedbacks, mediated by either post-translational modifications or *de novo* synthesized negative regulators, can lead to a transient reactivation or overshoot, but cannot fully restore output signaling (Figure 4A,B). Only partial reactivation, proportional to the strength of the feedback, can be brought about by a negative feedback loop, but a complete signaling reactivation can never occur [169]. 

System analyses of network adaptations highlight three ways in which treatment with a targeted inhibitor can be followed by a complete recovery, or even overshoot, of pathway signaling within a range of inhibitor doses [169]. First, when there are at least two connection routes, activating and inhibitory, from an inhibited upstream drug target to the downstream output, creating a feedforward loop in addition to the other connection route. A negative feedforward loop is found for instance in tumor cells with mutant RAS that activates both the RAF/MEK/ERK cascade and the p38 pathway, which in turn can inhibit ERK (Figure 4C,D). Second, when there is a crosstalk among two pathways, inhibition of one of them can favor the activation of the other. This may occur, for instance, when the output protein of the inhibited pathway exerts a negative feedback to a kinase at the crosstalk point, or further upstream. Inhibition of the output protein will thus reduce the negative feedback on the other pathway, favoring its activation. Third, complete reactivation of a signaling pathway may result from an increase in kinase dimerization induced by some kinase inhibitors, as described above for RAF inhibitors. The inhibitor-induced kinase dimerization can cooperate with inhibitor-mediated alleviation of negative feedback, bringing complete restoration or overshoot of signaling activity known as paradoxical activation by an inhibitor [169]. 

## 11. Signaling Network Models Can Predict Drug Sensitivity

Modeling the dynamics of signaling pathways to drug perturbations can predict cell type-specific responses to small-molecule therapeutics and prioritize primary drug targets and their combinations. Cell type-specific dynamic logic models of signaling networks were built for 14 colorectal cancer cell lines based on a large-scale signaling perturbation screening, encompassing 43 different perturbations that included 5 ligands that stimulate different receptors and 7 small-molecule kinase inhibitors [176]. Simulated signaling dynamics correlated with some drug sensitivities. A drug combination predicted to overcome resistance to MEK inhibitors by co-blockade of GSK3 was validated experimentally, thereby suggesting an advantage of simulating the dynamic signaling responses to drugs over static genotype data. 

A dynamic model of the estrogen (E) receptor (ER)-induced proliferation of MCF-7 breast cancer cells was built to predict responses to endocrine therapy [177]. This ODE model described the ER binding of its ligand E2, the facilitation of ER degradation by fulvestrant, the main interactions of ER signaling, the cell cycle machinery, including the transcription factor c-Myc, and cell proliferation as functions of retinoblastoma 1 (RB1) phosphorylation and the current number of cells. Experimental data on the abundance of key proteins, proliferation, and endocrine therapy treatments over a 7-day course were used for model calibration. Although oversimplified, this model was able to predict the responses to the combination treatment of E2 deprivation (endocrine therapy) and the drug Palbociclib that inhibited Cdk4/6 kinase [177]. Another ODE model described the transitions among different estrogen sensitivity phenotypes in breast cancer, known as sensitive, hypersensitive, and independent, aimed at optimizing sequential and intermittent endocrine treatments [178]. 

Immune checkpoint inhibitors (ICIs) greatly enhanced cancer treatment, yet many patients are intrinsically resistant to anti-programed cell death protein 1 (anti-PD1) and anti-cytotoxic T-lymphocyte-associated protein 4 (anti-CTLA4) therapies or they become resistant after initial response. A logic network model encompassing not only PD1 and CTLA4 immune checkpoints, but also three other inhibiting checkpoints (T-cell immunoglobulin and immunoreceptor tyrosine-based inhibition motif (TIGIT), lymphocyte activation gene 3 (LAG3), and T-cell immunoglobulin and mucin domain-containing protein 3 (TIM3)) and three activating checkpoints (inducible T-cell costimulatory (ICOS), cluster of differentiation 226 (CD226), and tumor necrosis factor receptors (TNFRs)) were built to explore efficient combinations of ICIs that can increase the sensitivity to immunotherapy [179]. First, this dynamic logic model recapitulated results of existing experimental studies of anti-PD1 and anti-CTLA4 therapies on T-cell activation. Then, the model suggested ICIs combinations predicted to be efficient with TIGIT as the most promising drug target. Future experimental immune checkpoint treatments may use results of dynamic models as the initial guide.

## 12. Patient-Specific Network Modeling

Models can be used to carry out patient-specific network simulations and to construct patient-specific dynamic biomarkers. 

Pioneering studies exploiting the systems-level models of apoptosis to predict the patient responses to chemotherapy were reported during the last decade. An ODEs-based model of the intrinsic apoptotic pathway, mediating the apoptotic effects of some chemotherapeutic agents, was shown to accurately predict the induction of apoptotic cell death based on the concentrations of the five key proteins pro-caspases 3 and 9, second mitochondria-derived activator of caspases (SMAC), apoptotic protease-activating factor 1 (APAF-1), and X-linked-inhibitor-of-apoptosis protein (XIAP) in HeLa tumor cells [180]. Patient-specific models of apoptosis execution, based on the concentrations of these molecules determined in samples of stage II and III colorectal tumors, showed a trend towards impaired apoptosis execution with advanced disease stage and correlated with disease relapse after adjuvant chemotherapy. They were the only significant predictor of patient outcome at multivariate analysis and outperformed predictors based on statistical analyses of apoptotic molecules [181]. Another computational model of the intrinsic apoptotic pathway, integrating data on the interaction of pro- and antiapoptotic BCL2 proteins, allowed to predict the sensitivity of colorectal cancer cell lines and tumor samples to chemotherapy [182]. Apoptotic models might be harnessed to select the better chemotherapeutic agent for each single tumor, based on the mechanisms of induction of apoptosis, or to predict the efficacy of targeted pro-apoptotic molecules [183]. Although this pioneering work led to remarkable advances in our understanding of chemoresistance resulting from the inability to elicit apoptosis, these models were limited to the mitochondria-dependent execution of apoptosis, whereas a number of major signaling pathways are frequently altered in cancer.

Logical models can be tailored to individual patient tumor samples by incorporating mutation, copy number variation, and expression data into the changes of the network node activities and state transition rates [184]. Stochastic simulations using a Monte Carlo kinetic algorithm have been used to compute the state probabilities. The resulting predictions of cancer phenotypes allow to formulate a proof of principle that clinical patient stratification can be obtained using personalized logical models [184].

Another recent study built a knowledge-based, logic model of the intrinsic and extrinsic apoptosis pathways, where logic states were formulated as ODEs to allow the use of continuous scales [185]. A microfluidic perturbation screening platform was used to test the apoptotic response to different drugs or drug combinations in cells collected from four pancreatic tumor biopsies and two tumor cell lines. The model represented the activation of caspase 3 as the final effector of apoptosis, after exposure of tumor cells to 1 or 2 drugs, chosen among 10 different compounds, including 7 kinase inhibitors, 1 cytokine, and 2 chemotherapeutic drugs. Dynamic models trained on data from cancer cell lines were used to simulate the effects of perturbations, allowing to predict effective drug combinations that were confirmed experimentally. Patient-specific dynamic models, trained on data collected from tumor biopsies, allowed to assess the heterogeneity of pancreatic cancers, highlighting differentially regulated signaling, especially in the PI3K-Akt pathway. Further development of similar computational models can make them suitable to determine personalized combinatorial treatments of cancer [186].

Other methods to define the best combination therapy according to the set of driver genes of an individual tumor are based on structural network controllability principles, which refer to the task of controlling the transition of complex networks from one state to another by intervening on a minimum set of nodes [187]. After applying network reconstruction methods on single tumor sample data obtained from breast and lung cancer datasets from The Cancer Genome Atlas (TCGA), a nonlinear structural network controllability method has been shown to outperform other existing synergistic combinatorial strategies in identifying clinical efficacious paired combinatorial drugs [188].

Individualized protein-protein interaction networks, which are developed from RNA-seq transcriptomic and genetic variants data, allow to discriminate among disease phenotypes [189]. 

Biochemical networks, in particular genome-wide metabolic networks, are often modeled through flux balance analysis, a constraint-based computational approach for predicting steady-state metabolic fluxes [190]. This approach can be integrated with metabolite concentrations and kinetic constants, yielding more realistic models [191,192,193]. Further combining this with multi-omics, kinetic, and thermodynamic information, personalized genome-scale models have been constructed, allowing to investigate the metabolic differences subtending different tumor phenotypes, such as resistance or sensitivity to radiation therapy, and to identify personalized therapeutic strategies for individual radiation-resistant patients [194].

The c-Jun N-terminal kinase (JNK) pathway is a MAPK cascade mediating apoptosis in response to different types of stress, including chemotherapeutic agents. JNK may undergo either a gradual activation in response to growth factors, promoting cell survival and proliferation, or an ultrasensitive, switch-like activation in response to stress, leading to apoptosis. The reconstruction of the JNK pathway in the SH-SY5Y neuroblastoma cell line identified a positive feedback from JNK to MKK7 as responsible for the ultrasensitive switch-like apoptotic response [195]. An ODEs-based model of JNK pathway was calibrated to fit experimental data obtained from the SH-SY5Y neuroblastoma cell line. Some predictions of the model, such as that ZAK overexpression would impair JNK activation, were experimentally confirmed, and the model was validated on different neuroblastoma cell lines and with different stressors. The model can be filled with data from a patient, instead of data from a cell line, to generate a patient-specific simulation of JNK pathway. For this purpose, data from gene expression profiles (as proxy to protein abundances) from a training cohort of neuroblastoma patients were used to generate a model simulation for each patient. The main output of the model, a curve describing the relationship between the amount of stress stimulus and the level of JNK activation (phosphorylation), can be characterized with three descriptors: maximal amplitude, activation threshold, and Hill exponent describing the ultrasensitivity of JNK response. An impaired ability to activate JNK is highlighted by high values of activation threshold and low values of maximal amplitude and Hill exponent. JNK response appeared to be increasingly impaired with increasing stage of the disease. After defining cutoff values for the output descriptors within the training cohort, able to maximally discriminate between good and poor prognosis patients, the prognostic value of the descriptors was confirmed on two validation patients’ cohorts, with the Hill exponent providing the greatest prognostic value. The Hill exponent was significantly associated with overall survival in both MYCN-amplified and non-MYCN-amplified patients’ subgroups and retained its independent prognostic role in multivariate analysis [195].

## 13. Conclusions

Despite major advances, cancer treatment remains an open challenge in many respects. Identifying the driver molecular alterations in a tumor is only a partial solution [4]. Although targeted therapies have produced outstanding results in specific tumor subtypes, treatments selected based on “agnostic” molecular alterations [24,196] produce modest results [197,198,199,200], highlighting the need for a systemic approach [201]. 

Relatively “simple” tumors, such as some cases of chronic myeloid leukemia or HER2-positive breast cancer, whose growth is driven by a key molecular alteration, can experience long-lasting remissions and likely cure with targeted treatments [202,203,204,205]. They may represent a proof of principle of the possibility to obtain long-term control, or even cure, of more complex tumors when (i) their key molecular alterations are correctly targeted, and (ii) network adaptations causing drug resistance are prevented by properly designated drug combinations. Describing and analyzing the intrinsic behavior of the biological processes that underlie tumor pathology, dynamical models will potentially allow to identify the key points for effective interventions with target drugs, substantially delaying or preventing resistance. 

Patient-specific network models require the incorporation of multiple types of data, including gene expression levels and key protein activities, post-translational modifications, and mutations that encompass the major driver alterations present in a single tumor. These dynamic models allow to perform patient-specific simulations of drug treatments and can help to identify the best drug or drug combination for an individual tumor. The personalized drug combinations can be validated in preclinical experiments, such as cell cultures, organoids, or patient-derived xenografts, and then tested in modern clinical trials.

Clinical development would require shifting from drug-centered clinical trials to patient-centered designs [26], allowing to administer a personalized drug combination to each patient. This could involve the use of multiple N-of-1 trials [206,207], or to adopt suitable biomarker strategy designs or other designs for predictive biomarker validation [208,209,210,211,212]. This may represent a future avenue for clinical and translational research. 

In summary, the techniques of systems biology allow to reconstruct the dynamics of biological processes. The information yielded is therefore potentially superior, both quantitatively and qualitatively, to that pertaining to single biomarkers or to panels of biomarkers [213]. We strongly endorse a wider application of systems biology methods in clinical and translational research, with a joint effort between scientists and clinicians, which should be the prerogative of academic institutions and cancer research centers and groups.
